# Short Dental Implants (≤8.5 mm) versus Standard Dental Implants (≥10 mm): A One-Year Post-Loading Prospective Observational Study

**DOI:** 10.3390/ijerph18115683

**Published:** 2021-05-26

**Authors:** Guillermo Pardo-Zamora, Antonio José Ortiz-Ruíz, Fabio Camacho-Alonso, José Francisco Martínez-Marco, Juan Manuel Molina-González, Núria Piqué-Clusella, Ascensión Vicente-Hernández

**Affiliations:** 1 Department of General Dentistry and Implants, Faculty of Medicine and Dentistry, University of Murcia, 30008 Murcia, Spain; ajortiz@um.es (A.J.O.-R.); fcamacho@um.es (F.C.-A.); josustus@msn.com (J.F.M.-M.); juanmamolinag@icloud.com (J.M.M.-G.); ascenvi@um.es (A.V.-H.); 2 Microbiology Section, Department of Biology, Healthcare and Environment, Faculty of Pharmacy and Food Sciences, Universitat de Barcelona (UB), Av Joan XXIII, 27-31, 08028 Barcelona, Spain; npique@ub.edu

**Keywords:** short implants, standard implants, partial edentulism, survival, implant stability, marginal bone level, dental implants

## Abstract

Background: Recent data have shown that short dental implants can be the preferred treatment in most of cases of posterior atrophic alveolar ridges, offering higher survival and lower complication rates than long implants. The survival rates, stability, and marginal bone level changes were compared between short implants (7 and 8.5 mm) and standard-length implants (≥10 mm). Methods: Prospective observational study in which adult patients requiring ≥1 osseointegrated implants to replace missing teeth were recruited consecutively. A clinical examination was performed on the day the definitive prosthesis was placed and after 6 and 12 months. Implant stability quotient (ISQ), marginal bone level (MBL) changes, and the correlation between these parameters and the characteristics of the implants were evaluated. Results: A total of 99 implants were inserted (47 short, 52 standard) in 74 patients. The 12-month survival rate was 100%. ISQ values showed a similar pattern for both types of implants. No correlation was found between ISQ changes after one year and MBL values, nor between the latter and the characteristics of the implants. Conclusions: With clinical treatment criteria, shorter implants (7 and 8.5 mm in length) can be just as useful as standard-length implants in atrophic alveolar ridges, demonstrating similar rates of survival, stability, and crestal bone loss.

## 1. Introduction

Partial edentulism in the posterior regions of the maxilla and the mandible is a common clinical condition. Removable partial dentures are badly tolerated because of their discomfort and instability stemming from the tongue’s own movements [1]. The ideal solution is to use implants that support a fixed prosthesis, with a length greater than 10 mm to ensure a good long-term prognosis [2].

Implants 10 mm or greater in length have traditionally been considered the standard length for implant therapy [2]. Long implants are generally thought to be more reliable than short implants as they have more surface area contact with the bone and lower crown-to-implant ratios, which is a more favorable feature [3], but recent systematic reviews on the use of short implants in the posterior region have concluded that there are no significant differences with regard to survival rate, crestal bone loss, and prosthesis survival rate compared to standard-length implants [2,4,5]. The definition of short implants is still controversial. The length for an implant to be considered as short has varied widely from 10 mm through ≤8.5 mm, ≤8 mm, <8 mm to 6 mm [4,6,7]. Moreover, implants with a length of ≤6.5 mm have been defined as extra-short [8]. They have been proposed as good alternatives for edentulous patients with posterior atrophic alveolar ridges to avoid alveolar bone augmentation techniques (bone grafts, maxillary sinus lift, dentoalveolar distraction, or block grafts), with high survival rates and lower complications than standard implants, although no consensus has yet been reached. Dental implantation in the posterior maxilla is usually challenging due to insufficient vertical bone volume, poor bone quality, reduced interarch space, limited visibility, and sinus pneumatization [1]. Although alveolar bone augmentation techniques have been successfully used with the purpose of creating the required bone volume for the placement of standard-size implants, such techniques usually have a high morbidity, can be difficult to perform, may bring some discomfort to the patient, and they increase the cost and the duration of the treatment [9,10]. Thus, short implants provide benefits in terms of reduced morbidity, less difficulty, shorter duration, and lower treatment costs [11,12].

However, the use of short dental implants may imply some technical difficulties, such as a greater precision is required to avoid any lesion to neurovascular structures and the risk of losing stability is greater in case of higher than average marginal bone loss over time [13].

Several studies have shown that most of the stress caused by occlusal loading is transferred to the cervical portion of the implant, while relatively little force reaches its apical portion, so the length of the implant is less critical than the diameter [14,15]. Moreover, the current surface treatments available, together with the advances in the implant designs, have helped to improve osseointegration, thus compensating for the reduction in implant length [13].

While previous research has shown that short implants with length less than 8 mm presented greater risk of failures [16], recent data have shown that short dental implants can be the preferred treatment in most cases, with high survival rates and lower complications [1,13,17].

Therefore, the objective of this prospective observational (1 year follow-up) was to test the hypothesis of no difference in the treatment outcome after the placement of short implants (7 and 8.5 mm) compared to standard length implants (≥10 mm) in daily clinical situations, in terms of survival rates, stability, as measured using Resonance Frequency Analysis (RFA), and marginal bone level changes.

## 2. Materials and Methods

### 2.1. Design and Characteristics of Participants

A prospective observational study was conducted at the University Dental Clinic of the School of Medicine and Dentistry of the University of Murcia (Department of Integrated Adult Dentistry and Master’s Course in Implants). From March 2015 to November 2017, adult patients were treated using Biomimetic Ocean implants with internal hex connections (Avinent Implant System^®^, Santpedor, Spain): standard (10 mm (29 implants), 11.5 mm (15 implants), 13 mm (7 implants), and 15 mm (1 implant)) and short (7 mm (17 implants) and 8.5 mm (30 implants)). Standard implants were used when there was a satisfactory length to harbor them; otherwise, short implants were placed.

The Biomimetic Ocean implants, with a modified calcium–phosphate surface, have a geometry that adapts to the biological architecture of the bone, promoting primary stability and preservation of the bone, and it has a positive angle platform switching to facilitate soft tissue adaptation. The implant is characterized by their biomimetic advanced surface, which is classified as moderately rough, and it has a surface topography that features macro-roughness, which is achieved through a physical process, and microporosity obtained through a chemical process whereby calcium and phosphorus are incorporated into the titanium oxide, thus creating an osteoconductive surface that enhances osseointegration [18].

Male and female adult subjects who met the following inclusion criteria were consecutively recruited: indication for the placement of one or more osseointegrated implants to replace missing teeth in native bone, having good oral hygiene (plaque index ≤25%, [19]), and no systemic disorders or habits that would contraindicate treatment with dental implants. Treatment with short or standard implants was provided based on native bone availability.

Exclusion criteria included having an active or serious infection at the site of implant placement, having a previous bone regeneration, having an uncontrolled systemic disease, smoking (more than 10 cigarettes/day), having a record of radiation treatment to the head and neck (within the 6 months prior to the inclusion), pregnancy.

The patients were informed about the treatment; they agreed to it and signed the written informed consent statement, agreeing to return to the clinic for all the study follow-up visits. Patients who did not comply with the visits and procedures were excluded from the study.

The study was conducted in accordance with the Declaration of Helsinki Ethical Principles and was approved by the Research Ethics Committee of the University of Murcia (ethical approval code: 1762/2017). Compliance with all applicable points of the STROBE guidelines was checked.

### 2.2. Methodology

Examinations were performed on the day the temporary restoration was placed, three months after loading, the day the definitive prosthesis was placed and 6 and 12 months after the latter was loaded. Intraoral periapical X-rays were obtained on the day of surgery (MBL1), the day the implant was loaded with the definitive prosthesis (MBL2), and 12 months after loading (MBL3). Implant stability was assessed following placement of the implant (implant stability quotient, ISQ1), at the start of the definitive prosthetic phase (ISQ2), and 12 months after loading (ISQ3).

#### 2.2.1. Surgical Procedure

All subjects underwent an initial comprehensive periodontal examination, including probing pocket depth (PPD), recession (REC), clinical attachment level (CAL), and bleeding on probing (BOP) on 6 sites per tooth in all teeth present, excluding third molars. All the implant placement procedures were performed by the same surgeon, in the same operating room, and under local anesthesia, in accordance with the implant manufacturer’s surgical protocol. Standard implants were used when there was a satisfactory length to harbor them; otherwise, short implants were placed. In a few cases where small bone defects were detected after implant insertion, autologous bone tissue particles mixed with collagenated porcine bone xenograft granules were inserted (Gen-Os, Osteobiol Tecnoss^®^ Dental s.r.l, Torino, Italy). The ISQ was determined before the cover caps were screwed on. As postoperative treatment, amoxicillin/clavulanic acid (Augmentin^®^ 875 mg + 125 mg every 8 h for 7 days, GSK, Madrid, Spain) and 0.12% chlorhexidine mouthwashes (Perio-Aid^®^, Dentaid, Barcelona, Spain) were prescribed together with Ibuprofen 600 mg (Norvectan^®^, Pharmacodynamic Applications Laboratory, SA Barcelona, Spain) in case of pain. Postoperative instructions for oral care and hygiene were given. The sutures were removed seven days after surgery.

#### 2.2.2. Prosthetic Procedure

Since placement, periods of 2 and 3 months were considered to achieve osseointegration for implants inserted in the mandible and for implants inserted in the upper jaw, respectively.

Between 8 and 12 weeks after implant placement, impressions were taken to make a screw-retained acrylic provisional. This provisional restoration was placed 2 weeks later, and then, it was modified until proper gingival contours were obtained, and the patient practiced good oral hygiene. Four months after the provisional restoration placement, impressions were taken for the definitive prosthesis; all single crowns were fixed using a titanium interface for ceramic, zirconia, or zirconia–ceramic crowns and monolithic–zirconia or zirconia–ceramic bridges in cases where there were two or more implants.

#### 2.2.3. Clinical Examinations

Clinical examinations that included the presence of bacterial plaque, probing pocket depth (PPD), and bleeding on probing (BOP) measurement on mesial, distal, vestibular, and lingual/palatal surfaces were performed on the day the temporary restoration was placed, three months after it was loaded, on the definitive prosthetic loading and 6 and 12 months follow-up. At each examination, dental cleaning was performed, and the instructions given for oral hygiene were reinforced in cases where there was a significant presence of bacterial plaque and/or bleeding.

#### 2.2.4. Radiographic Measurements

Intraoral periapical X-rays (Kodak^®^ ultra-speed film DF-58. Kodak^®^. Rochester, NY, USA) were used to assess implant crestal bone levels on the day of surgery (MBL1), the day the implant was loaded with the definitive prosthesis (MBL2), and 12 months after loading (MBL3), measuring the vertical distance from the implant shoulder to the most coronal part of implant–bone contact at mesial and distal. The mean of these measurements was used as a reference point for the marginal bone level of each implant at the beginning of the study and at the follow-up time-points on the day the definitive prosthesis was loaded and 12 months after loading. Marginal bone level changes were calculated by comparing these mean values over time. To minimize error and to standardize the technique, a holding system with a customized positioner (Rinn^®^ system, Dentsply Sirona, York, Pa, USA) with self-curing silicone bite blocks for the teeth in the region adjacent to the implant was used. A researcher and expert in radiology from the Radiology Dept., Universidad de Murcia, performed all radiographic measurements in a blind manner using imaging analysis software (ImageJ64 1.47 v, Wayne Rasband, Bethesda, MD, USA).

#### 2.2.5. Stability Assessment

Primary ISQs were measured immediately after implant placement by recording the RFA using Osstell ISQ^®^ (Gamlestadsvägen, Göteborg, Sweden), as per the manufacturer’s instructions. These values were recorded after placement of the implant (ISQ1), at the start of the definitive prosthetic phase (ISQ2) and 12 months after loading (ISQ3). To do so, a SmartPeg^®^ (Ostell AB, Gothenburg, Sweden) was manually screwed to the implant using a torque wrench at a force of 10 Ncm2, as recommended by the manufacturer. With the SmartPeg^®^ in place, an Osstell Mentor^®^ (Ostell AB, Gothenburg, Sweden) probe with an antenna perpendicular to the SmartPeg^®^ was used to test the implant in all four possible directions: vestibular, lingual, mesial, and distal, until a continuous beep was heard, which indicated that the reading had been taken with the result appearing directly on the device’s screen. Every implant was measured twice from different angles: around 90 degrees apart and parallel to the ridge. The average of the two measurements was calculated.

### 2.3. Variables

The main outcome variables included implant stability (ISQ), marginal bone level (MBL), and implant survival. Implants were considered to have survived if they were at their site of placement and functioned at follow-up control visits. Any implants that were lost spontaneously or extracted after placement were recorded as failures. Sex, age, implant diameter, type of bone, and type of prosthesis were considered as factors or covariates.

### 2.4. Statistical Analysis

In the descriptive analysis, values were expressed as mean ± standard deviation (SD), absolute values, and percentages.

The implant was considered as the statistical unit. Accepting an alpha risk of 0.05 and a beta risk of 0.2 in a two-sided test, a minimum of 32 subjects per groups are necessary to detect a significant change in the marginal bone level (MBL) greater than or equal to 0.15 units with a standard deviation of 0.20. A drop-out rate of 10% was anticipated (“Sample size and power calculator”. https://www.imim.es/ofertadeserveis/software-public/granmo/index.html, accessed on 15 January 2015). 

Qualitative variables were compared using contingency tables and Fisher’s exact test or Pearson’s chi-square test.

The Kolmogorov–Smirnov normality test and Levene’s test for equality of variances were used for quantitative variables. Between-group comparisons were made using the Student’s *t*-test when there was normality and equality of variances and the Mann–Whitney test when there was not.

ISQ and MBL values on the implant placement (T1), the day the definitive prosthesis was loaded (T2), and 12 months after prosthetic loading (T3) were compared using the Friedman repeated measurements test and, in cases where differences were significant, Tukey’s comparison test was used. In order to prevent small differences in the initial ISQ and MBL values (day of surgery) from having an effect on potential differences between the groups, instead of using absolute values, the increments in the values at the two study timepoints were used. These were obtained by subtracting T1 values from the T2 and T3 values. Thus, the Δ ISQ2-ISQ1 value was obtained by subtracting the ISQ value on the day surgery was performed from the ISQ value on the prosthetic loading; the Δ ISQ3-ISQ2 value was obtained by subtracting the ISQ value on the day the prosthesis were loaded from the value at the 12-month follow-up. The Δ MBL2-MBL1 value and Δ MBL3-MBL2 value were similarly obtained.

To determine whether there was any correlation between the Δ ISQ values and the Δ MBL values according to the different variables (implant length, diameter, type of bone and type of prosthesis), Pearson’s correlation test was performed. In order to establish any possible interaction of factors sex and age in the behavior of ISQ and MBL in short and standard implants, we have performed a two-way analysis of variance.

The statistical analysis was performed using the SigmaStat 3.5 statistical package (Systat Software Inc., Point Richmond, CA, USA). A *p*-value < 0.05 was considered to be statistically significant.

## 3. Results

### 3.1. Patient and Implant Characteristics

A total of 74 patients were included: 35 men (47.29%) and 39 women (52.70%), ranging in age from 22 to 82 years (mean age: 47.98 years). The mean age of the patients in the group with short implants was 51.76 ± 9.46 years and 44.68 ± 10.94 years in the standard implant group (*p* = 0.008). The characteristics of the participants in the study, demographic data and implant characteristics are shown in Table 1. A total of 99 implants were placed in accordance with the implant manufacturer’s surgical protocol; 47 short implants in 33 patients and 52 standard implants in 41 patients. With regard to implant site, 28 implants were placed in the maxilla and 19 were placed in the mandible in the group with short implants; 44 (93.61%) of these were in the molar and premolar regions. In the group with standard implants, 33 were placed in the maxilla and 19 were placed in the mandible; their distribution was more homogeneous: 24 in the anterior region (46.16%) and 28 in the posterior region (53.84%). With respect to the diameter of the implants, the most common measurement in both groups was 4 mm (40.42% for the short implants and 46.16% for the standard implants). In the short implant group, single crowns were loaded in 40.42% of the cases and in the remainder (59.58%), 2-, 3-, and 4-unit bridges were loaded.

**Figure 1:**
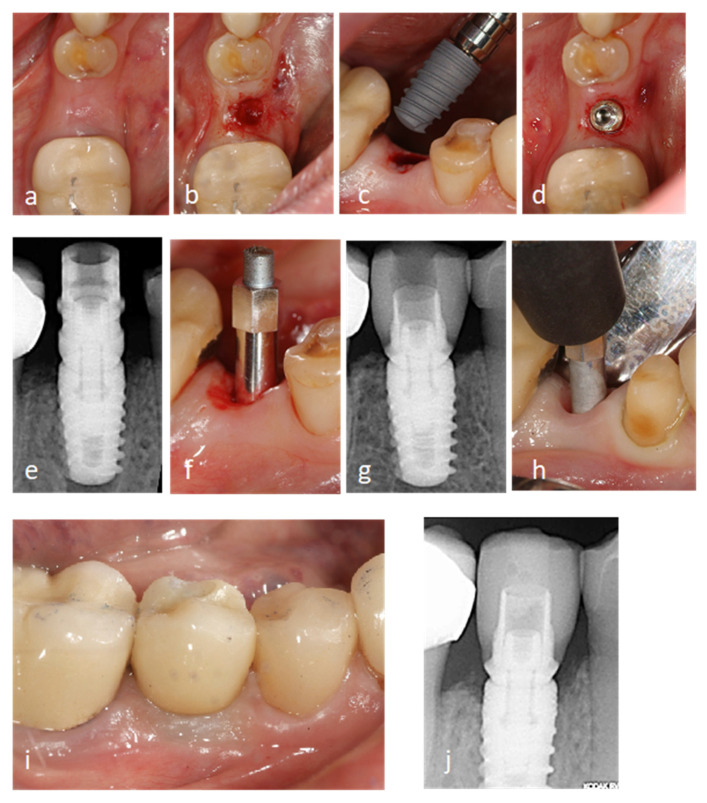
Clinical photographs and X-rays belonging to the short implants patient group (7 and 8.5 mm). Photographs taken during the surgery procedure of a 7 mm implant being placed (**a**–**d**), standardized periapical X-ray and implant stability quotient (ISQ) value measurement immediately after the implant placement (**e**,**f**), standardized periapical X-ray and ISQ value measurement day the definite prosthesis was loaded (**g**,**h**), Clinical photograph and standardized periapical X-ray 12 months follow-up (**i**,**j**).

Figure 1 shows a clinical case that is representative of the short implants group. The photographs and radiographs illustrate the set of steps followed on the day the implant surgery was performed (Figure 1a–f), the day the definitive prosthesis was loaded at the end of the provisionalization period (Figure 1g,h), and at the 12-month follow-up (Figure 1i,j). The same two-stage surgical protocol was followed in every case, with a waiting time before loading of 2 months for the mandible and 3 months for the upper maxilla. After the surgery, clinical controls were performed at 15 days and one month after performing the second surgery. Clinical examinations were performed at 15 days after the surgery and one month prior to performing the second surgery. In two implants in the upper maxilla (at the site of the second premolar and the first molar), slight painless mobility was observed when the healing abutment was placed, so it was decided to wait another two months before taking impressions for the temporary prosthesis. All the implants were initially loaded with a provisional prosthesis for progressive loading and molding of the soft tissues for at least one month before taking impressions for the definitive prostheses.

No failures were reported for the study implants during the 12 months after loading, achieving a 12-month survival rate of 100% in both groups.

### 3.2. Assessment of Implant Stability (ISQ)

**Figure 2:**
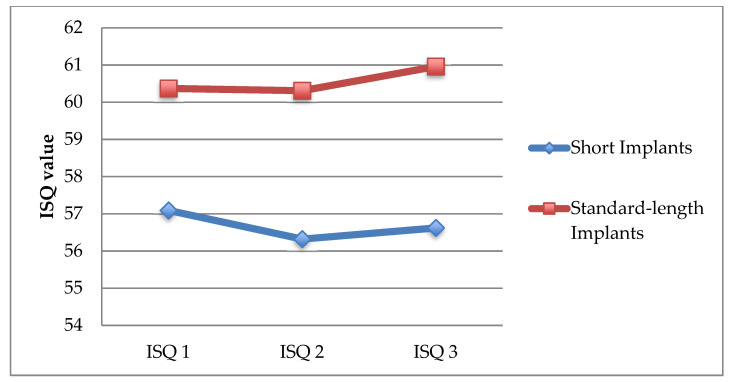
Changes in mean ISQ values of patients with standard implants (≥10 mm) and short implants (7 and 8.5 mm).

Figure 2 shows the ISQ measurements made on the implant placement (ISQ1), the prosthetic loading (ISQ2), and at 12 months follow-up (ISQ3). ISQ values showed a similar pattern for both short and standard implants.

ISQ values showed a decrease from time of surgery to the time of loading the definitive prostheses of −0.745 ± 2.192 units for short implants and −0.057 ± 2.796 units for standard implants, but they subsequently showed a significant increase at 12 months after loading the prosthesis that were 0.298 ± 1.876 units for short implants (*p* = 0.014) and 0.654 ± 1.781 units for standard implants (*p* = 0.043) (Table 2).

### 3.3. Assessment of Changes in Marginal Bone Level (MBL)

**Figure 3:**
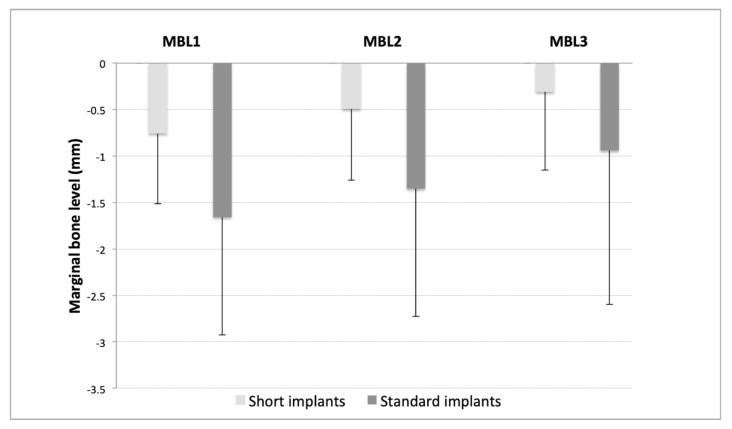
Average marginal bone levels (mm) in patients with standard implants (≥10 mm) and short implants (7 and 8.5 mm).

We evaluated MBL at mesial and distal in both implant groups on the day of surgery (MBL1), implant loading (MBL2), and 12 months after loading (MBL3). In both groups, a continuous reduction of MBL values was observed since the day of surgery to 12 months post-loading (Figure 3). In the short implants group, values were from −0.76 ± 0.76 mm (MBL1) to −0.49 ± 0.77 mm (MBL2) and −0.31 ± 0.84 mm (MBL3). In the group with standard implants, values were from −1.66 ± 1.27 mm (MBL1) to −1.35 ± 1.37 mm (MBL2) and −0.94 ± 1.66 mm (MBL3).

The bone loss of the short implants from time of surgery to time of loading the definitive prosthesis was similar to that of the standard implants (−0.263 ± 0.244 mm vs. –0.305 ± 0.272 mm; *p* = 0.324). There was still bone loss from time of loading the prothesis to 12 months after, but it was significantly less for short implants than for standard implants (−0.184 ± 0.191 mm vs. −0.412 ± 0.588 mm; *p* = 0.004) (Table 3).

### 3.4. Correlation between Changes in ISQ and Marginal Bone Level (MBL) Changes According to Length, Diameter, Position, and Type of Prosthesis

No correlation was found between ISQ changes after one year and marginal bone levels from baseline to 12 months after loading.

There was no correlation between the increase in ISQ and bone loss/gain in relation to the implants, regardless of their length. A slightly positive correlation was found between the Δ MBL2-MBL1 and Δ MBL2-MBL3 values in the short implants (0.664, *p* = 0.00000037).

No correlation was found between the increase in ISQ and bone loss/gain in relation to the implants, regardless of their diameter. However, we did find a weak correlation in wide diameter implants between Δ ISQ1-ISQ2 and Δ ISQ2-ISQ3 values (cc: 0.539, *p* = 0.000194) and Δ MBL1-MBL2 and Δ MBL2-MBL3 values (cc: 0.467; *p* = 0.0016).

Neither did we find any correlation between ISQ and bone loss/gain in relation to implant site, nor between ISQ and bone loss/gain in relation to the type of prosthesis (whether single crowns were supported or whether they were included/splinted onto a bridge of two or more implants).

### 3.5. Interaction between Factors (Sex and Age) with Type of Impant (Short and Standard) in ISQ and MBL Changes

There was not a statistically significant interaction between sex and implant length in ΔISQ2-ISQ1 (*p* = 0.052), ISQ3-ISQ2 (*p* = 0.781), MBL2-MBL1 (*p* = 0.908), and MBL3-MBL2 (*p* = 0.414).

There was not a statistically significant interaction between age and implant length in ΔISQ2-ISQ1 (*p* = 0.483), ISQ3-ISQ2 (*p* = 0.642), MBL2-MBL1 (*p* = 0.775), and MBL2 (*p* = 0.932).

## 4. Discussion

In implant planning, the selection of a length for the dental implants normally depends on the quantity and quality of the available bone at the implant site, with longer implants providing more primary stability and a more favorable and appropriate distribution of occlusal forces due to the larger contact surface between the implant and bone tissue [20]. Nonetheless, there is substantial evidence showing that short implants can be predictable, with survival rates similar to those of standard-length implants [21].

Short implants appeared in the market to be a feasible alternative to the long implants, with techniques of bone augmentation such as maxillary sinus lift, vertical regeneration with xenografts or blocks, distraction osteogenesis, or inferior alveolar nerve lateralization [11]. For many years, procedures such as sinus elevation with or without bone grafting have been considered the treatments of choice in atrophic posterior ridges, leading to high survival rates [22]. Despite this, the placement of long implants with bone regeneration techniques is associated with higher costs, longer times of treatment, increased morbidity, and the need for greater professional experience, with the consequent increase in the number of complications such as pain, inflammation, and potential infections [1]. In our study, the use of short or standard implants was based solely on bone availability. We have used both implant lengths to rehabilitate single and multiple crowns in the anterior and posterior areas in both the maxilla and the mandible. This study has demonstrated that the use of short implants 7 and 8.5 mm in length has no effect on survival, marginal bone loss, or primary/secondary stability of the implant compared to implants that are 10 or more millimeters in length at one year of follow-up. Our results are consistent with those reported by de Jung et al. (2018), Tolentino da Rosa de Souza et al. (2018), and Anitua and Alkhraisat (2019) [17,21,23]. In the consensus report from the 6th ITI Conference held in 2018, Jung et al. (2018) reported that the survival rates for both types of implants were similar after periods of 1 to 5 years [21]. Tolentino da Rosa de Souza et al. (2018) concluded that over a one-year follow-up, the survival rate, marginal bone loss, prosthetic failures, and surgical complications for short implants were similar to those for long implants in posterior single crowns [23], while Anitua and Alkhraisat (2019) found that the survival rate for short implants was 93.3% after a follow-up of 15 years [17].

The present study was conducted in daily clinical conditions where standard-length implants were used when enough bone was available, and when there was not, short implants were used instead. The results of this study support the use of short implants with a design and type of surface that allow outcomes similar to those of standard-length implants.

The demographic characteristics of the patients and the distribution of implants did not reveal statistically significant differences between the two groups.

The survival rate of the implants under study was 100% in both groups, which was similar to the results obtained in a number of studies [5,17,23]. However, other studies have concluded that short implants have a lower rate of survival than standard-length implants [24,25]. This discrepancy in the literature may be attributed to several factors, such as implant site, bone thickness and density, type of prosthesis and connection, loading protocols, implant design and surface, and others. The implant surface is a critical factor in the survival of short implants, with significantly greater success of implants with moderately rough surfaces, as there is higher surface area contact between the implant and the bone tissue and, therefore, greater resistance to the traction forces acting on the implant [26,27]. In our study, we used implants with a surface treatment that combines a shot-blasting process followed by an electrochemical (anodized) treatment that modifies the surface microtopography, causing porosity in the titanium oxide and, at the same time, allowing chemical elements (calcium and phosphorus) to be incorporated, providing a roughness to the surface that enables greater implant–bone contact [18]. In 2004, a systematic review concluded that implant surface geometry is a major determinant in the performance of short implants [28]. Griffin and Cheung in 2004 recognized maximized implant surface area as the most contributing factor to the high success rate of short implants [29]. Gentile et al., in their study, found high survival rates with rough surface implants and a two-stage surgical protocol in the placement of short implants, similar to those obtained in our study [30].

At present, the criteria used to define success in implant dentistry are absence of mobility, absence of peri-implant radiolucency in periapical radiographs, absence of pain or paraesthesia in the area of the implant, absence of peri-implant infections or suppuration, absence of bacterial plaque and bleeding, minimal amount of keratinized mucosa, vertical bone loss less than 0.2 mm annually following the implant’s first year of service, and a successful rate of 85% at the end of a five-year observation period and 80% at the end of a ten-year period [31,32].

With regard to stability, RFA has been widely used in both experimental and clinical studies over the past few years and has demonstrated a good correlation between the ISQ values achieved and the degree of rigidity at the implant–bone interface [33]. The ISQ values at baseline can be considered a criterion for implant success; along these lines, in 2004, Bischof et al. established that an ISQ value ≥54 can be considered as an indicator of success [34]. Other authors have proposed values ranging from an ISQ of 49 [35] to an ISQ of 60 [36]. In our study, no significant differences in the ISQ values on the day of surgery were found between the groups (long implants: ISQ1 of 57.09 ± 12.37, short implants: 60.37 ± 8.72), demonstrating that short implants can achieve a primary stability similar to that of standard-length implants. These values are similar to those obtained in the studies by Zix et al. in 2005 [37] (52.5 ± 7.9) and by Östman et al. in 2006 [38] (from 62.6 ± 0.0 to 67.4 ± 8.6). Primary stability is gradually replaced by secondary stability at the implant–bone interface and remains constant four weeks after surgery.

In our study, we found elevated ISQ values on the day of surgery followed by a slight decrease at the time of loading the definitive prosthesis, with no significant differences between groups. The similar values between both groups may be related to the design of the short implant used, conical and with a high number of threads, which allowed high primary stability to be achieved in most cases, together with greater implant–bone surface area contact due to the high roughness of its surface, which compensated for the shorter length. The decrease in ISQ values at the time of loading corresponds to the healing and osseointegration process of the implant, as shown in published studies [39]. At 12 months after loading, we found a significant increase in ISQ values with respect to the time of placement of the prostheses in both groups, without significant differences between both groups.

Regarding crestal bone loss, the stability of marginal bone levels is essential for the long-term success of implants. Standardized periapical radiographs are usually used to measure gain/loss of marginal bone tissue in the interproximal level [40]. We analyzed both mesial and distal crestal bone levels on the day of surgery for implant placement, on the day the definitive prosthesis was loaded, and at 12 months after loading. At the time of surgery, all implants for both groups were placed crestally or subcrestally, with negative values, and without significant differences. From the time of surgery to the loading of the implants with the definitive prosthesis, we found slight marginal bone loss in both groups, without significant differences between the groups. However, from the time the definitive prosthesis is loaded until 12 months after, bone continues to be lost, although the loss is significantly lower for short implants than for long ones (−0.184 ± 0.191 mm vs. −0.412 ± 0.588 mm; *p* = 0.004). Marginal bone stability, particularly around 7 mm implants, is especially critical due to the limited length. The results obtained in our study are similar to those obtained in different randomized controlled trials [41,42]. The reasons for which longer implants are found to have greater marginal bone loss are not well understood, but we can speculate about possible causes: (a) short implants lose less bone, since they are placed in basal bone tissue due to resorption processes, while long implants are placed in alveolar bone, being more prone to bone remodeling processes; (b) overheating during surgery as deeper osteotomies are performed, which is a phenomenon that has been described in the literature [2,43]. In our study, we followed the implant supplier’s drilling protocol, using new drills for each procedure and external and internal irrigation. Another possible cause for the greater marginal bone loss in the long implant group is the fact that a higher percentage of the implants were loaded with single crowns (>60%), whereas in the short implants group, less than half of the implants were loaded with single crowns. This fact can lead us to think that splinted implants achieve better load distribution, thus preventing greater crestal bone loss caused by occlusal overload [44]. However, there has been no corroboration by other studies regarding any significant effects of crown to implant size on implant survival or marginal bone loss, nor has it been possible to demonstrate that splinting has any significant effect on implant survival or bone loss [45].

Regarding the type of prosthesis connection, a recent systematic review on short implants concluded that there is significantly greater marginal bone loss in external connection implants compared to internal connection implants [46]. The implants we used in our study have internal connections with platform reduction (“platform switching”), which in several studies has been associated with lower crestal bone loss around the implants [47].

No correlation was observed between the changes in ISQ values and MBL changes. Although implant stability increased from the time the prostheses were loaded, the majority of implants had gradual marginal bone loss, without correlation between marginal bone changes and stability. Likewise, we found no positive correlation between changes in ISQ values and changes in MBL based on length, diameter, position, and type of prosthesis. These results are similar to those found in other studies that have assessed the stability of implants over time without finding any significant effects of marginal bone loss and changes in ISQ values [48].

Regarding possible associated biological or technical complications, during the performance of our study, no complications were reported. In a systematic review performed by de N Dias et al. (2019), the most frequently reported biological complication associated with short implants was the occurrence of paresthesia, which was also reported with conventional implants [13]. It should be noted that technical complications are more frequent with short implants and must be closely monitored during the 3 first years post-loading [22]. However, there are not statistically significant differences in biological and technical complications between both types of implants placed in regenerated sites [13,22].

Among the strengths of our study, we have confirmed in routine clinical conditions and with treatment planning criteria that the usefulness of short implants is comparable to standard implants, due to their characteristics such as rough surfaces or design.

As limitations, we believe that a study conducted over several years would have made it more robust. However, the investigation is ongoing, and results after 5 years will soon be reported. We also believe that a larger sample of patients would validate our results.

## 5. Conclusions

The results of this prospective observational study have shown that with clinical treatment planning criteria, shorter implants (7 and 8.5 mm in length) can be useful as standard-length implants in atrophic alveolar ridges, demonstrating similar rates of survival, stability, and crestal bone loss. Therefore, short implants might be a preferable choice to bone augmentation, particularly in posterior mandibles, since treatment is faster, cheaper, and associated with less morbidity. However, long-term studies are necessary before making reliable recommendations.

The results seem very encouraging, considering the 100% implant survival and the lower bone loss around short implants. However, this is an observational and not an interventional study, and the two groups vary significantly regarding implant location, implant diameter, and type of restoration. Moreover, the choice of implant length was not arbitrary. Therefore, interpretation of comparative results should be done with caution.

## Figures and Tables

**Table 1:** Patients and implant characteristics. Homogeneity of the study groups in terms of the demographic characteristics and dental implants distribution (Student’s t and Pearson’s χ^2^ tests).

Characteristics	Short Implants Group (*n* = 33 pt and *n* = 47 ix)	Standard Implants Group (*n* = 41 pt and *n* = 52 ix)	*p*-Value
Patients (*n* = 74)			
Age: mean ± SD	51.76 ± 9.46	44.68 ± 10.94	0.008 (*t*-test)
Gender: *n* (%)			0.776 (χ^2^)
Male	15 (45.46)	20 (48.79)	
Female	18 (54.54)	21 (51.21)	
Implants (*n* = 99)			
Maxilla/Mandible: *n* (%)			0.849 (χ^2^)
Maxilla	28 (59.57)	33 (63.46)	
Mandible	19 (40.43)	19 (36.54)	
Anterior/Posterior: *n* (%)			<0.001 (χ^2^)
Anterior	3 (6.39)	24 (46.16)	
Posterior	44 (93.61)	28 (53.84)	
Diameter: *n* (%)			0.001 (χ^2^)
3.5 mm^2^	0 (0.00)	13 (25.00)	
4.0 mm^2^	19 (40.42)	24 (46.16)	
4.5 mm^2^	13 (22.67)	6 (11.53)	
5.0 mm^2^	15 (31.91)	9 (17.31)	
Type of restoration: *n* (%)			0.036 (χ^2^)
Single crowns	19 (40.42)	32 (61.53)	
Fixed partial prosthesis	28 (59.58)	20 (38.47)	

SD = standard deviation; pt = patients; ix = implants; *t*-test = Student’s *t*-test; χ^2^ = Pearson’s χ^2^ test.

**Table 2:** Comparison of ISQ increases between short and standard implants at the three follow-up time points.

Time of Follow-Up	Short Implants (*n* = 47) Mean ± SD	Standard Implants (*n* = 52) Mean ± SD	*p*-Value
Δ ISQ2-ISQ1	−0.745 ± 2.192	−0.057 ± 2.796	=0.316 (MW)
Δ ISQ3-ISQ2	0.298 ± 1.876	0.654 ± 1.781	=0.336 (MW)
*p*-value	=0.014 (Friedman + Tukey)	=0.043 (Friedman + Tukey)	

The Δ ISQ2-ISQ1 value was obtained by subtracting the ISQ value on the day of surgery from the ISQ value on the prosthetic loading; the Δ ISQ3- ISQ2 value was obtained by subtracting the ISQ value on the day the prosthesis were loaded from the value 12 months follow-up. MW: Mann–Whitney test.

**Table 3:** Comparison of the increases of the vertical distance from the implant shoulder to the most coronal aspects of implant–bone contact (MBL) (medial, mesial, and distal) between both study groups at the two follow-up time points.

Time of Follow-Up	Short Implants (*n* = 47) Mean ± SD	Standard Implants (*n* = 52) Mean ± SD	*p*-Value
Δ MBL2-MBL1	−0.263 ± 0.244	−0.305 ± 0.272	=0.324 (MW)
Δ MBL3-MBL2	−0.184 ± 0.191	−0.412 ± 0.588	=0.004 (MW)
*p*-value	=0.009 (Friedman + Tukey)	=0.889 (Friedman + Tukey)	

The Δ MBL2-MBL1 value was obtained by subtracting the bone loss value on the day of surgery from the value on the prosthetic loading; the Δ MBL3-MBL2 value was obtained by subtracting the bone loss value on the day the prosthesis was loaded from the value at 12 months. MW: Mann–Whitney test.

## Data Availability

Data available on request due to restrictions. The data presented in this study are available on request from the corresponding author.
